# Supplementary material for: Symmetry in the open-system dynamics of quantum correlations
Source: Sci Rep. 2017 Aug 21;7:8367. doi: 10.1038/s41598-017-08457-1 (PMC5566315; doi:10.1038/s41598-017-08457-1)
Supplement: Supplementary file 1 — Supplementary material to: Symmetry in the open-system dynamics of quantum correlations [file 41598_2017_8457_MOESM1_ESM.pdf]

Supplementary material to: Symmetry in the open-system dynamics of quantum correlations

Henri Lyyra[1,*], Göktuğ Karpat[2,3], Chuan-Feng Li[4,5], Guang-Can Guo[4,5], Jyrki Piilo[1], and Sabrina Maniscalco[1,6]

[1]Turku Center for Quantum Physics, Department of Physics and Astronomy, Turku, FIN-20014 Turun yliopisto, Finland
[2]Faculdade de Ciências, UNESP - Universidade Estadual Paulista, Bauru, SP, 17033-360, Brazil
[3]Faculty of Arts and Sciences, Department of Physics, Izmir University of Economics, Izmir, 35330, Turkey
[4]CAS Key Laboratory of Quantum Information, University of Science and Technology of China, Hefei, 230026, People's Republic of China
[5]Synergetic Innovation Center of Quantum Information and Quantum Physics, University of Science and Technology of China, Hefei, 230026, People's Republic of China
[6]Centre for Quantum Engineering, Department of Applied Physics, Helsinki, P.O. Box 11000, FIN-00076 Aalto, Finland
[*]hesaly@utu.fi

Bit-flip channel

One-qubit bit-flip channel can be represented by Kraus operators $K_1' = \sqrt{p}\,\sigma_1$, $K_2' = \sqrt{q}I$. The dynamics of an arbitrary X state under one-sided bit-flip channels can be written as

$$
\begin{aligned}
\Phi_p^{B,U}(X) &= \begin{pmatrix} q\rho_{11}+p\rho_{33} & 0 & 0 & q\rho_{14}+p\rho_{23}^* \\ 0 & q\rho_{22}+\rho_{44}p & q\rho_{23}+p\rho_{14}^* & 0 \\ 0 & q\rho_{23}^*+p\rho_{14} & q\rho_{33}+\rho_{11}p & 0 \\ q\rho_{14}^*+p\rho_{23} & 0 & 0 & q\rho_{44}+\rho_{22}p \end{pmatrix}, \\
\Phi_p^{B,L}(X) &= \begin{pmatrix} q\rho_{11}+p\rho_{22} & 0 & 0 & q\rho_{14}+p\rho_{23} \\ 0 & q\rho_{22}+\rho_{11}p & q\rho_{23}+p\rho_{14} & 0 \\ 0 & q\rho_{23}^*+p\rho_{14}^* & q\rho_{33}+\rho_{44}p & 0 \\ q\rho_{14}^*+p\rho_{23}^* & 0 & 0 & q\rho_{44}+\rho_{33}p \end{pmatrix}.
\end{aligned} \tag{S1}
$$

By using equation (3) of the main article, we get as the concurrences of the output states

$$
\begin{aligned}
C\big(\Phi_p^{B,U}(X)\big) &= 2\max\Big\{0,\, |\rho_{14}p+\rho_{23}^*q| - \sqrt{(\rho_{22}p+\rho_{44}q)(\rho_{33}p+\rho_{11}q)},\, |\rho_{14}^*q+\rho_{23}p| - \sqrt{(\rho_{11}p+\rho_{33}q)(\rho_{44}p+\rho_{22}q)}\Big\}, \\
C\big(\Phi_p^{B,L}(X)\big) &= 2\max\Big\{0,\, |\rho_{14}^*p+\rho_{23}^*q| - \sqrt{(\rho_{33}p+\rho_{44}q)(\rho_{22}p+\rho_{11}q)},\, |\rho_{14}^*q+\rho_{23}^*p| - \sqrt{(\rho_{11}p+\rho_{22}q)(\rho_{44}p+\rho_{33}q)}\Big\}.
\end{aligned} \tag{S2}
$$

Due to the form of the solutions it is not as simple to solve analytically, when the decay of entanglement is symmetric. Still we can find a set of sufficient conditions for symmetric entanglement decay:

$$
(\rho_{14} \in \mathbb{R} \text{ or } \rho_{23} \in \mathbb{R}) \text{ and } (\rho_{22} = \rho_{33} \text{ or } \rho_{11} = \rho_{44}) \tag{S3}
$$

Bit-phase-flip channel

One-qubit bit-phase-flip channel can be represented by Kraus operators $K_1' = \sqrt{p}\,\sigma_2$, $K_2' = \sqrt{q}I$. The dynamics of an arbitrary X state under one-sided bit-phase-flip channels can be written as

$$
\begin{aligned}
\Phi_p^{P,U}(X) &= \begin{pmatrix} q\rho_{11}+p\rho_{33} & 0 & 0 & q\rho_{14}-p\rho_{23}^* \\ 0 & q\rho_{22}+\rho_{44}p & q\rho_{23}-p\rho_{14}^* & 0 \\ 0 & q\rho_{23}^*-p\rho_{14} & q\rho_{33}+\rho_{11}p & 0 \\ q\rho_{14}^*-p\rho_{23} & 0 & 0 & q\rho_{44}+\rho_{22}p \end{pmatrix}, \\
\Phi_p^{P,L}(X) &= \begin{pmatrix} q\rho_{11}+p\rho_{22} & 0 & 0 & q\rho_{14}-p\rho_{23} \\ 0 & q\rho_{22}+\rho_{11}p & q\rho_{23}-p\rho_{14} & 0 \\ 0 & q\rho_{23}^*-p\rho_{14}^* & q\rho_{33}+\rho_{44}p & 0 \\ q\rho_{14}^*-p\rho_{23}^* & 0 & 0 & q\rho_{44}+\rho_{33}p \end{pmatrix}.
\end{aligned} \tag{S4}
$$

By using equation (3) of the main article, we get as the concurrences of the output states

$$
\begin{aligned}
C\big(\Phi_p^{P,U}(X)\big) &= 2\max\Big\{0,\, |\rho_{23}^*q-\rho_{14}p| - \sqrt{(\rho_{22}p+\rho_{44}q)(\rho_{33}p+\rho_{11}q)},\, |\rho_{14}^*q+\rho_{23}p| - \sqrt{(\rho_{11}p+\rho_{33}q)(\rho_{44}p+\rho_{22}q)}\Big\}, \\
C\big(\Phi_p^{P,L}(X)\big) &= 2\max\Big\{0,\, |\rho_{23}^*q-\rho_{14}^*p| - \sqrt{(\rho_{33}p+\rho_{44}q)(\rho_{22}p+\rho_{11}q)},\, |\rho_{14}^*q-\rho_{23}^*p| - \sqrt{(\rho_{11}p+\rho_{22}q)(\rho_{44}p+\rho_{33}q)}\Big\}.
\end{aligned} \tag{S5}
$$
